# Hemoglobin concentration is associated with the incidence of metabolic syndrome

**DOI:** 10.1186/s12902-021-00719-4

**Published:** 2021-03-19

**Authors:** Sunyue He, Hongxia Gu, Jie Yang, Qing Su, Xiaoyong Li, Li Qin

**Affiliations:** 1 Department of Endocrinology, Xinhua Hospital Chongming Branch, School of Medicine, Shanghai Jiaotong University, 25 Nanmen Road, Shanghai, 202150 China; 2 Department of Endocrinology, Xinhua Hospital, School of Medicine, Shanghai Jiaotong University, 1665 Kongjiang Road, Shanghai, 200092 China

**Keywords:** Epidemiology, Hemoglobin level, Metabolic syndrome, Metabolic syndrome components

## Abstract

**Background:**

An association between hemoglobin and metabolic syndrome (MetS) has been reported. However, the relationships between hemoglobin and individual MetS components remain unclear. Therefore, we investigated these associations at baseline and at the 3-year follow-up.

**Methods:**

We enrolled 9960 middle-aged and elderly subjects (6726 women and 3234 men) and performed a 3-year follow-up cohort study. All subjects completed a questionnaire and underwent anthropometric measurements and laboratory tests. Logistic regression models were developed to assess the association between hemoglobin and MetS and its components.

**Results:**

MetS was present in 45.1% of women and 41.4% of men at baseline. The hemoglobin concentration was positively correlated with SBP, DBP, TGs, WC, FPG, insulin, HOMA-IR, BMI and uric acid (*p* < 0.05). The mean hemoglobin concentration was higher in subjects with hypertension, high TGs, abdominal obesity or elevated FPG (*p* < 0.01). At follow-up, elevated hemoglobin correlated with an increased incidence and ORs for MetS, high TGs, low HDL-c, hyperuricemia and NAFLD but not abdominal obesity, BP or FPG in women. Increased hemoglobin corresponded with an increased incidence and ORs for MetS, abdominal obesity, low HDL-c, hyperuricemia and NAFLD but not BP, high TGs or FPG in men.

**Conclusions:**

Hemoglobin may play a role in predicting new-onset MetS in both women and men. Hemoglobin was notably correlated with future risk of high TGs, low HDL-c, hyperuricemia, and NAFLD among women and abdominal obesity, low HDL-c, hyperuricemia, and NAFLD among men.

## Background

Metabolic syndrome (MetS) is defined as a clustering of common cardiovascular risk factors, such as hypertension, hypertriglyceridemia, low high-density lipoprotein cholesterol (HDL-c), visceral obesity, and impaired fasting glucose levels [1]. MetS is known to be associated with an increased risk of type 2 diabetes and cardiovascular disease (CVD), which is the primary cause of morbidity and mortality and results in a substantial economic burden on society [2–4]. A recent study conducted among middle-aged and elderly adults in China revealed an unexpectedly high estimated prevalence of MetS (18.4% according to the ATP III criteria, 34.0% according to the revised ATP III criteria, and 26.9% according to the International Diabetes Federation (IDF) criteria), and MetS may be even more common due to the continuous increase in obesity [5]. Considering its status as an emerging epidemic and its impact, early detection of individuals at high risk for MetS would help prevent the associated diabetes-related and cardiovascular complications.

Various cross-sectional studies have demonstrated an association between high hemoglobin concentration and the prevalence of MetS [6–9]. Furthermore, this positive association was detected in several cohort studies [10, 11]. Recently, a population-based study revealed that the hemoglobin concentration was associated with an increased risk of MetS in men [12]. However, the association between the hemoglobin level and MetS and its individual components remains unclear. In addition, evidence from large-scale samples regarding the relationship between hemoglobin and MetS is scarce.

Therefore, we conducted a follow-up study to verify the association between hemoglobin and the prevalence of MetS and to further assess the predictive ability of hemoglobin for new-onset MetS in individuals stratified by sex in a Chinese rural cohort.

## Methods

### Study population and design

**Fig. 1:**
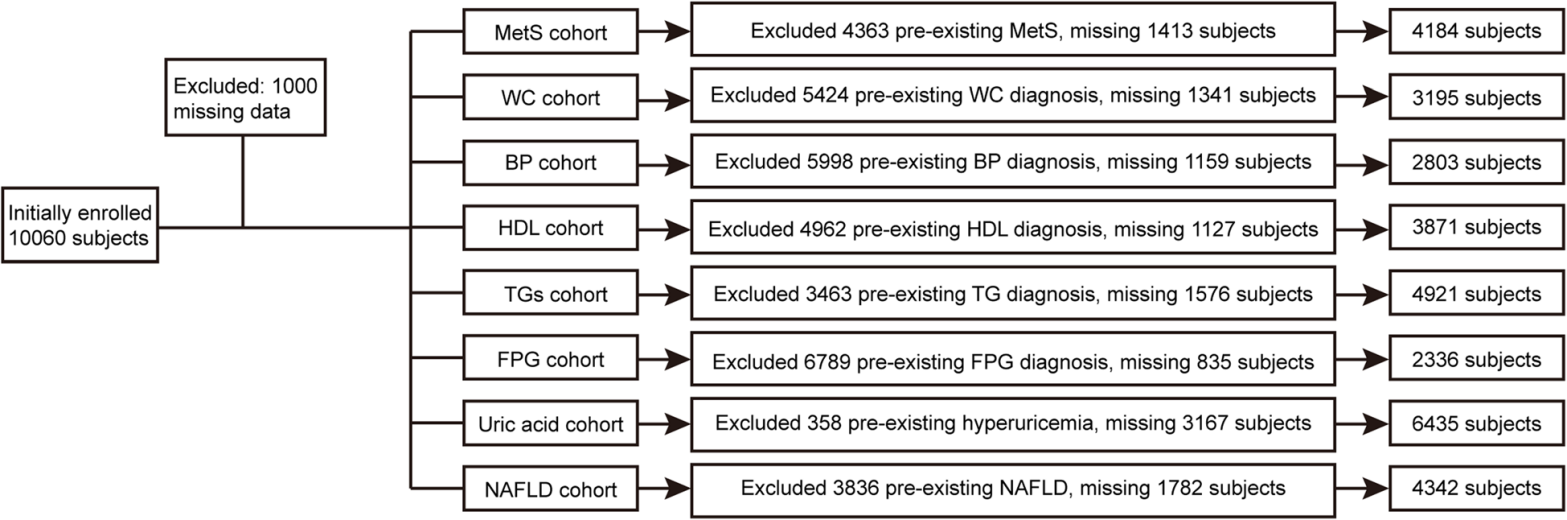
Study design flow diagram

The study is a part of the national survey of Risk Evaluation of Cancer in Chinese Diabetic Individuals, a longitudinal (REACTION) study [13], which was a population-based sub-cohort study conducted amongst adults aged 40 years and older, from 2011 to 2014. The study design and methods have been described previously in detail [13–15]. The data presented in this article are based on the survey of subsamples from the Chongming District, Shanghai, China. We recruited 10,060 subjects in total, and they were all approved to participate in the first phase of our research. Individuals with missing data for hemoglobin and metabolic variables were excluded. Finally, 9960 eligible subjects were enrolled in the study and underwent a 3-year follow-up. The study design flow diagram is presented in Fig. 1.

### Data collection

A standardized questionnaire was used by certified medical workers via interviews to collect essential information such as demographic characteristics (e.g., age, sex, education), alcohol consumption, smoking status, and disease history. Blood pressure (BP) were measured on the right arm of participants after sitting down and rested quietly > 5 min. The measurements were taken three times in 5 min intervals, and the mean value was used in the statistical analysis. Waist circumference (WC) was measured at the midpoint between the lowest costal margin and the lateral iliac crest with the participant in a standing position.

### Laboratory methods

After an overnight fast of 8–10 h, peripheral venous blood samples were obtained for measurements of various factors. Fasting plasma glucose (FPG) were measured by the glucose oxidase method (ADVIA-1650 Chemistry System, Bayer). Hemoglobin A1c (HbA1c) was assessed by high-performance liquid chromatography (BIO-RAD, D10). Fasting insulin was tested by radioimmunoassay (Linco Research). Serum low-density lipoprotein cholesterol (LDL-c), HDL-c, total cholesterol (TC), triglycerides (TGs), serum creatinine (SCr) and uric acid (UA) were measured in fasting blood samples using an automated biochemical instrument (Hitachi 7080). Hemoglobin was measured by the cyanmethemoglobin method [16]. The homeostasis model assessment of insulin resistance (HOMA-IR) and beta-cell function (HOMA-β) was calculated on the basis of the equation described by Matthews et al. [17]. Glomerular filtration rate (GFR) was estimated using the equation described by Liu et al. [18].

### Definitions

MetS was defined based on the updated National Cholesterol Education Program Expert Panel on Detection, Evaluation and Treatment of High Blood Cholesterol in Adults (NCEP-ATPIII) for Asian Americans according to the presentation of 3 or more of the following components [19] [1]: BP ≥130/85 mmHg and/or current use of antihypertensive medications in both sexes [2]; serum HDL cholesterol < 1.03 mmol/l for men or < 1.29 mmol/l for women [3]; serum TGs ≥1.7 mmol/l in both sexes [4]; WC ≥90 cm for men or ≥ 80 cm for women; and [5] FPG ≥5.6 mmol/l and/or previous diagnosis of type 2 diabetes in both sexes. The validity of this definition was confirmed previously. Hyperuricemia was defined as serum uric acid> 420 μmol/L in males and > 360 μmol/L in females or a history of anti-UA medication use [20]. Nonalcoholic fatty liver disease (NAFLD) was diagnosed according to the criteria described by the Chinese Liver Disease Association [21].

### Statistical analysis

The normality of continuous variables was evaluated by the Kolmogorov-Smirnov test. Skewed variables such as FPG and TGs were presented as medians (interquartile ranges), and continuous variables were presented as the means ± standard deviations. Categorical variables were reported as the numbers (percentages). Unpaired Student’s t-test and chi-square tests were used to analyze statistical differences among the study participant’s characteristics. The relationship between hemoglobin levels and HOMA-IR was analyzed by one-way ANOVA. Partial Spearman’s correlations were performed to evaluate the associations between various related parameters and hemoglobin levels. Logistic regression models were used to estimate the odds ratios (ORs) and confidence intervals (CIs) of individual components of MetS for every quartile of hemoglobin compared to the lowest quartile. All statistical analyses were performed with SPSS 22.0 (SPSS Inc.; Chicago, IL). All statistical tests were two-sided and a *P*-value < 0.05 was considered to indicate statistical significance.

## Results

The study population included 6726 women and 3234 men with mean ages of 55.3 ± 7.9 and 57.7 ± 7.6 years, respectively. MetS was present in 45.1% of women and 41.4% of men. The clinical characteristics and lifestyle factors of subjects with and without MetS are presented in Table 1. Both women and men with MetS were clearly older than participants without MetS. Baseline BMI, WC, BP, FPG, HbA1c, insulin level, LDL-c, TGs, hemoglobin, UA and NAFLD prevalence were notably higher, while HDL-c and eGFR were lower in men and women with MetS than in those without MetS (Table 1). Education level was significantly different between the MetS group and non-MetS group in women but not in men. Lifestyle factors such as smoking and alcohol consumption habits did not differ significantly between participants with and without MetS.

**Table 1:** Clinical and laboratory characteristics of the study population in subjects with and without the MetS at baseline

Characteristics	Men (*n* = 3234)		*P* value	Women (*n* = 6726)		P value
	MetS not present (*n* = 1895/58.6%)	MetS present (1339/41.4%)		MetS not present (*n* = 3694/54.9%)	MetS present (*n* = 3032/45.1%)	
Age, year	57.29 ± 7.66	58.31 ± 7.46	*P* < 0.001	53.38 ± 7.95	57.54 ± 7.31	P < 0.001
BMI, kg/m^2^	23.75 ± 6.13	26.85 ± 4.51	P < 0.001	23.17 ± 4.19	26.43 ± 9.99	P < 0.001
WC, cm	84.26 ± 7.79	93.96 ± 7.22	P < 0.001	78.44 ± 8.47	88.23 ± 7.88	P < 0.001
FPG, mmol/L	5.8 (5.4–6.4)	6.3 (5.9–7.2)	P < 0.001	5.5 (5.2–5.9)	6.1 (5.7–6.9)	P < 0.001
HbA1c, %	5.7 (5.4–6.0)	6.0 (5.6–6.5)	P < 0.001	5.7 (5.4–6.0)	6.0 (5.6–6.4)	P < 0.001
SBP, mmHg	130.21 ± 17.81	139.94 ± 18.09	P < 0.001	123.08 ± 17.30	135.66 ± 17.64	P < 0.001
DBP, mmHg	80.69 ± 9.69	85.77 ± 9.68	P < 0.001	76.96 ± 9.47	81.9 ± 9.62	P < 0.001
HDL-c, mmol/L	1.27 ± 0.32	1.02 ± 0.25	P < 0.001	1.36 ± 0.32	1.13 ± 0.25	P < 0.001
LDL-c, mmol/L	2.61 ± 0.72	2.49 ± 0.75	P < 0.001	2.60 ± 0.77	2.68 ± 0.80	P < 0.001
TC, mmol/L	4.57 ± 0.93	4.55 ± 1.08	*P* = 0.73	4.59 ± 1.03	4.81 ± 1.05	P < 0.001
TGs, mmol/L	1.16 (0.87–1.54)	2.10 (1.53–2.97)	P < 0.001	1.05 (0.81–1.37)	1.91 (1.37–2.63)	P < 0.001
NAFLD, n (%)	438 (23.1)	889 (66.4)	P < 0.001	857 (23.2)	2017 (66.5)	P < 0.001
Hemoglobin, g/L	156.63 ± 11.51	159.62 ± 11.43	P < 0.001	135.68 ± 11.56	139.58 ± 10.57	P < 0.001
Smoking, n (%)	906 (49)	615 (46.6)	*P* = 0.181	45 (1.3)	31 (1.1)	*P* = 0.441
Drinking, n (%)	945 (51.2)	723 (54.8)	*P* = 0.046	319 (9.1)	229 (7.9)	*P* = 0.095
Tea, n (%)	673 (36.6)	579 (44.6)	P < 0.001	766 (21.5)	610 (20.6)	*P* = 0.38
UA, μmol/L	282 ± 64.81	325.8 ± 74.83	P < 0.001	207.30 ± 49.36	247.20 ± 61.25	P < 0.001
Insulin, pmol/L	4.9 (3.4–6.7)	7.9 (5.8–10.5)	P < 0.001	5.8 (4.3–7.5)	8.7 (6.5–11.5)	P < 0.001
eGFR, mL/min/1.73 m^2^	128.76 ± 24.89	122.76 ± 28.34	*P* < 0.001	128.76 ± 24.89	121.09 ± 23.24	P < 0.001
Heart rate, b/min	76.97 ± 12.65	78.62 ± 11.716	P < 0.001	80.34 ± 11.61	81.44 ± 11.97	P < 0.001
Education, n (%)			*P* = 0.719			P < 0.001
Literate or semi-literate	37 (2.0)	20 (1.5)		123 (3.4)	187 (6.2)	
Primary school	311 (16.5)	215 (16.1)		548 (14.9)	757 (25.1)	
Junior high school	954 (50.7)	665 (49.9)		1830 (49.9)	1392 (46.2)	
High school	403 (21.4)	307 (20.3)		1037 (28.3)	610 (20.2)	
College (or) and above	175 (9.3)	127 (9.5)		130 (3.5)	70 (2.3)	

Note: The values are presented as the mean ± standard deviation (median with interquartile range) or Number (proportions). Abbreviations: BMI, body mass index; WC, waist circumference; FPG, fasting plasma glucose; HbA1c, hemoglobin A1c; DBP, diastolic blood pressure; SBP, systolic blood pressure; HDL-c, high-density lipoprotein cholesterol; LDL-c, low-density lipoprotein cholesterol; TC, total cholesterol; TGs, triglycerides; UA, uric acid; eGFR, estimated glomerular filtration rate

Partial Spearman’s rank correlation showed that the baseline hemoglobin concentration was significantly and positively correlated with SBP(*r* = 0.144 in men, *r* = 0.163 in women), DBP(*r* = 0.250 in men, *r* = 0.241 in women), TGs(*r* = 0.162 in men, *r* = 0.139 in women), WC(*r* = 0.185 in men, *r* = 0.147 in women), FPG(*r* = 0.109 in men, *r* = 0.152 in women), insulin(*r* = 0.062 in men, *r* = 0.082 in women), HOMA-IR(*r* = 0.054 in men, *r* = 0.088 in women), BMI (*r* = 0.087 in men, *r* = 0.067 in women) and UA(*r* = 0.048 in men, *r* = 0.105 in women), while the hemoglobin concentration was not correlated with HDL-c or HOMA-β.

**Fig. 2:**
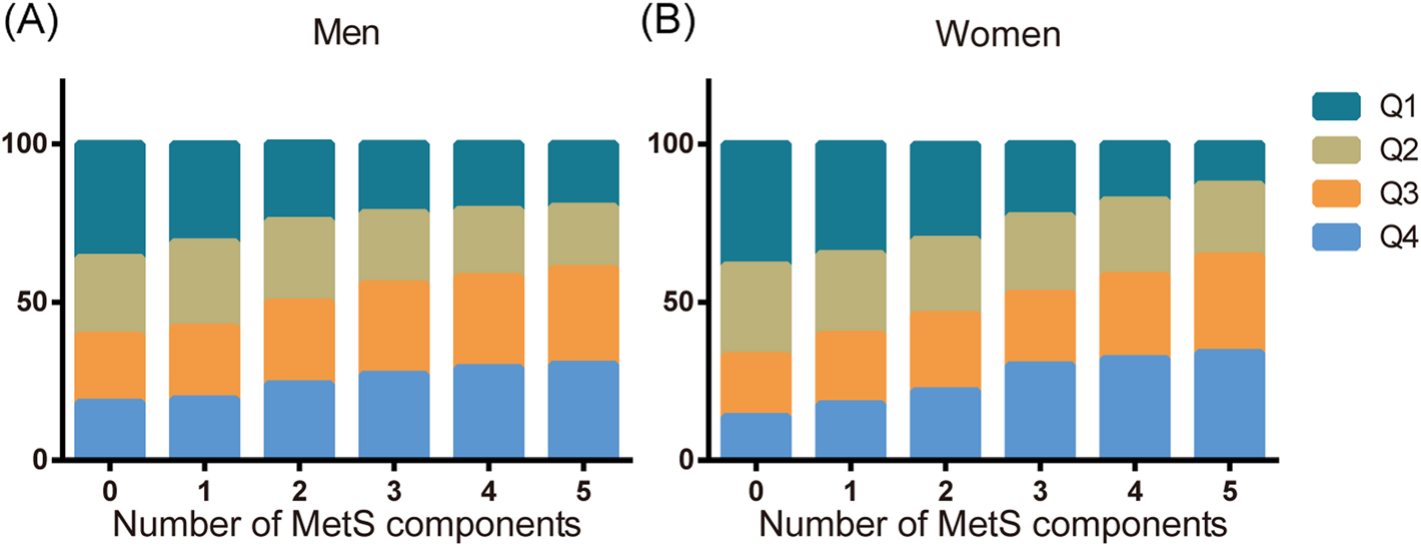
The proportion of individuals in baseline hemoglobin concentration quartiles according to different numbers of MetS components

Figure 2 shows the proportion of individuals in baseline hemoglobin concentration quartiles according to different numbers of MetS components. As the number of MetS components increased, the proportion of individuals in the first quartile decreased from 38.5 to 12.9%, and the proportion of individuals in the fourth quartile increased from 13.6 to 33.9% among women. The same trend was observed in men: as the number of MetS components increased, the proportion of individuals in the first quartile decreased from 36.1 to 19.8%, and the proportion of individuals in the fourth quartile increased from 18.1 to 30.2%.

**Fig. 3:**
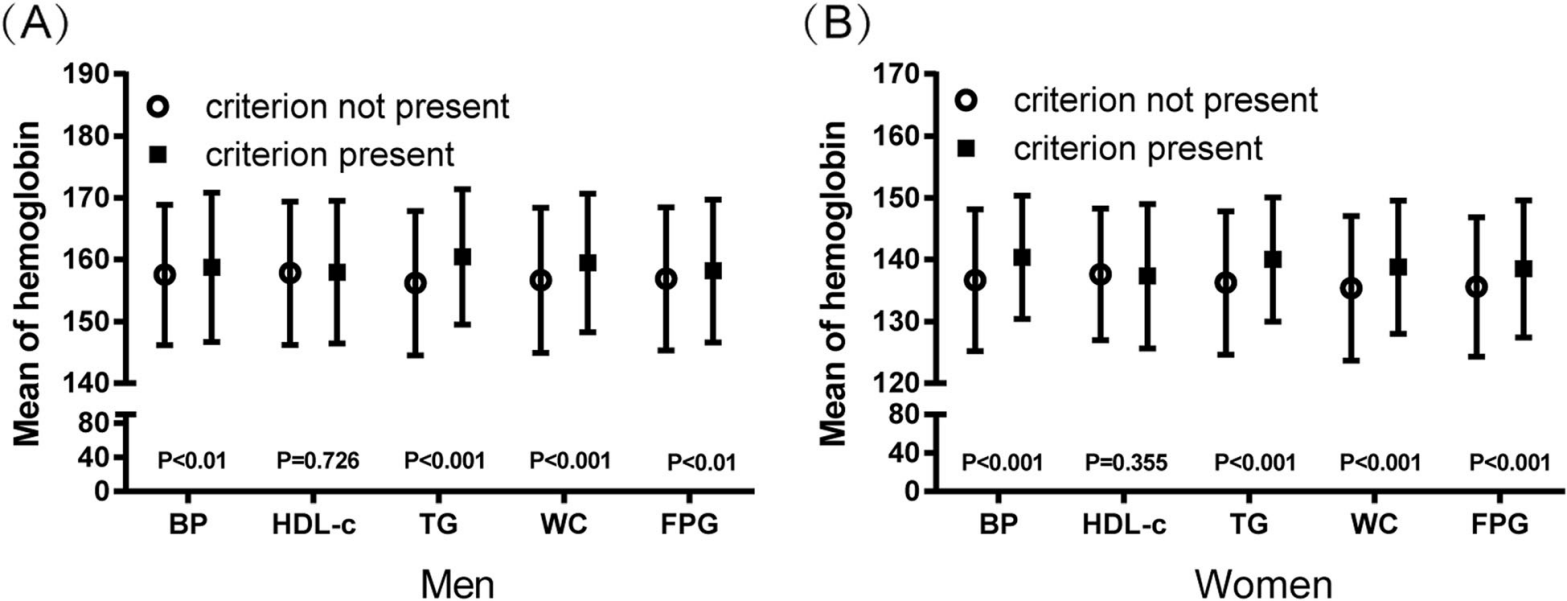
Mean hemoglobin values and individual MetS components. Data are presented as mean ± SD

Figure 3 shows the mean values of hemoglobin in subjects with and without each MetS component. Subjects with hypertension, high TGs, abdominal obesity or elevated glucose were more likely to have higher hemoglobin levels. However, there was no significant difference in individuals with low HDL-c. Next, we evaluated the risk of developing MetS/its components according to serum hemoglobin concentration. Table 2 shows the prevalence as well as ORs (95% CI) for each component of MetS and MetS itself at follow-up in the different hemoglobin quartiles among men. Increased levels of hemoglobin corresponded with an increase in the incidence of and ORs for MetS, abdominal obesity and low HDL-c but not BP, high TGs or FPG. A greater range of hemoglobin was correlated with the development of MetS in men than in women. Compared with the first quartile of hemoglobin, participants in the fourth quartile of hemoglobin had significantly higher ORs for MetS [1.53 (1.10–2.12), *P* < 0.05], abdominal obesity [1.61 (1.15–2.26), P < 0.05] and low HDL-c [1.57 (1.01–2.44), P < 0.05]. The adjusted ORs for MetS and abdominal obesity were slightly reduced after adjusting for age, education, smoking and alcohol consumption habits in men. After further adjusting for SBP, DBP, FPG, TGs, HDL-c, UA, eGFR and NAFLD, the adjusted OR of abdominal obesity was 1.65 (95% CI 1.12–2.43, P < 0.05).

**Table 2:** Odds ratio and 95% CI of each component of MetS at 3-year follow-up according to baseline hemoglobin quartiles in men

MetS	Q1 (< 150 g/L)	Q2 (150–157 g/L)	Q3 (158–163 g/L)	Q4 (≥164 g/L)
	*n* = 314	*n* = 351	*n* = 367	*n* = 360
Incidence, n (%)	8 5 (24.9)	90 (25.6)	115 (31.3)	121 (33.6)
Model 1	1 (Ref)	1.04 (0.74–1.45)	1.37 (0.99–1.91)	**1.53 (1.10–2.12)** ^*^
Model 2	1 (Ref)	1.05 (0.74–1.49)	1.35 (0.96–1.90)	**1.50 (1.06–2.11)** ^*^
Model 3^†^	1 (Ref)	0.84 (0.57–1.25)	1.03 (0.70–1.53)	1.11 (0.75–1.65)
WC	Q1 (< 151 g/L)	Q2 (151–157 g/L)	Q3 (158–164 g/L)	Q4 (≥165 g/L)
	*n* = 347	*n* = 324	*n* = 322	*n* = 311
Incidence, n (%)	87 (25.1)	96 (29.6)	107 (33.2)	109 (35.0)
Model 1	1 (Ref)	1.26 (0.90–1.77)	**1.49 (1.06–2.08)** ^*^	**1.61 (1.15–2.26)** ^*^
Model 2	1 (Ref)	1.28 (0.90–1.82)	**1.60 (1.13–2.26)** ^*^	**1.75 (1.23–2.49)** ^*^
Model 3^‡^	1 (Ref)	1.21 (0.83–1.75)	**1.58 (1.09–2.28)** ^*^	**1.65 (1.12–2.43)** ^*^
BP	Q1 (< 150 g/L)	Q2 (150-156g/L)	Q3 (157-162g/L)	Q4 (≥163 g/L)
	*n* = 168	*n* = 160	*n* = 175	*n* = 185
Incidence, n (%)	88 (52.4)	68 (42.5)	91 (52.0)	105 (56.8)
Model 1	1 (Ref)	0.67 (0.44–1.04)	0.99 (0.65–1.51)	1.19 (0.78–1.82)
Model 2	1 (Ref)	0.67 (0.43–1.04)	1.04 (0.67–1.61)	1.28 (0.83–2.00)
Model 3^§^	1 (Ref)	0.76 (0.47–1.23)	1.09 (0.67–1.74)	1.24 (0.76–2.00)
TGs	Q1 (< 150 g/L)	Q2 (150-156g/L)	Q3 (157-163g/L)	Q4 (≥164 g/L)
	*n* = 379	n = 360	*n* = 378	*n* = 361
Incidence, n (%)	68 (17.9)	60 (16.7)	76 (20.1)	79 (21.9)
Model 1	1 (Ref)	0.92 (0.62–1.34)	1.15 (0.80–1.65)	1.28 (0.89–1.84)
Model 2	1 (Ref)	0.90 (0.61–1.33)	1.07 (0.74–1.57)	1.20 (0.83–1.75)
Model 3^¶^	1 (Ref)	0.94 (0.60–1.45)	0.96 (0.62–1.48)	1.23 (0.80–1.89)
HDL-c	Q1 (< 151 g/L)	Q2 (151-157g/L)	Q3 (158-164g/L)	Q4 (≥165 g/L)
	*n* = 384	*n* = 395	*n* = 415	*n* = 406
Incidence, n (%)	36 (9.4)	48 (12.2)	54 (13.0)	57 (14.0)
Model 1	1 (Ref)	1.33 (0.84–2.09)	1.43 (0.92–2.24)	**1.57 (1.01–2.44)** ^*^
Model 2	1 (Ref)	1.19 (0.75–1.91)	1.37 (0.87–2.16)	1.51 (0.96–2.38)
Model 3^††^	1 (Ref)	1.10 (0.66–1.82)	1.18 (0.72–1.94)	1.23 (0.75–2.03)
FBG	Q1 (< 149 g/L)	Q2 (149-156g/L)	Q3 (157-163g/L)	Q4 (≥164 g/L)
	*n* = 149	*n* = 134	*n* = 141	*n* = 137
Incidence, n (%)	48 (38.1)	43 (32.1)	53 (37.6)	42 (30.7)
Model 1	1 (Ref)	0.77 (0.46–1.28)	0.98 (0.60–1.61)	0.72 (0.43–1.20)
Model 2	1 (Ref)	0.85 (0.50–1.44)	0.98 (0.58–1.66)	0.74 (0.43–1.27)
Model 3^‡‡^	1 (Ref)	0.88 (0.47–1.66)	1.00 (0.54–1.89)	0.76 (0.39–1.49)

Abbreviations: CI, confidential interval; WC, waist circumference; DBP, diastolic blood pressure; SBP, systolic blood pressure; FPG, fasting plasma glucose; HDL-c, high-density lipoprotein cholesterol; TGs, triglycerides; eGFR, estimated glomerular filtration rate; the use of bold emphasis and * are to mark the statistically significant indicators

Model 1 is unadjusted;

Model 2 is adjusted for age, education, smoking and drinking;

Model 3^†^ is adjusted for age, education, smoking, drinking, WC, SBP, DBP, FPG, HDL-c, TGs, uric acid, NAFLD and eGFR

Model 3^‡^ is adjusted for age, education, smoking, drinking, SBP, DBP, FPG, HDL-c, TGs, uric acid, NAFLD and eGFR

Model 3^§^ is adjusted for age, education, smoking, drinking, WC, FPG, HDL-c, TGs, uric acid, NAFLD and eGFR

Model 3^¶^ is adjusted for age, education, smoking, drinking, WC, SBP, DBP, FPG, HDL-c, uric acid, NAFLD and eGFR

Model 3^††^ is adjusted for age, education, smoking, drinking, WC, SBP, DBP, FPG, TGs, uric acid, NAFLD and eGFR

Model 3^‡‡^ is adjusted for age, education, smoking, drinking, WC, SBP, DBP, HDL-c, TGs, uric acid, NAFLD and eGFR

The associations of hemoglobin quartiles with individual MetS components at the 3-year follow-up in women are shown in Table 3. Increased levels of hemoglobin were correlated with an increase in the incidence and ORs for MetS, high TGs and low HDL-c but not abdominal obesity, BP, or FPG. There were increasing trends in the incidence and ORs for MetS, high TGs and low HDL-c from the first to the fourth quartiles of hemoglobin. Compared with the lowest quartile of hemoglobin, subjects in the highest quartile of hemoglobin had significantly higher ORs for MetS [1.44 (1.16–1.78), *P* < 0.05], abdominal obesity [1.56 (1.23–1.98), P < 0.05] and low HDL-c [1.43 (1.08–1.88), P < 0.05]. The adjusted ORs for high TGs were slightly reduced after adjusting for age, education, smoking and alcohol consumption habits but increased slightly for MetS and low HDL-c in women.

**Table 3:** Odds ratio and 95% CI of each component of MetS at 3-year follow-up according to baseline hemoglobin quartiles in women

MetS	Q1 (< 130 g/L)	Q2 (130-136g/L)	Q3 (137-142g/L)	Q4 (≥143 g/L)
	*n* = 716	*n* = 654	*n* = 703	*n* = 719
Incidence, n (%)	242 (33.8)	218 (33.3)	281 (40.0)	304 (42.3)
Model 1	1 (Ref)	0.98 (0.78–1.23)	**1.30 (1.05–1.62)** ^*^	**1.44 (1.15–1.78)** ^*^
Model 2	1 (Ref)	0.93 (0.74–1.18)	**1.32 (1.05–1.66)** ^*^	**1.47 (1.17–1.84)** ^*^
Model 3^†^	1 (Ref)	0.88 (0.67–1.18)	1.24 (0.95–1.62)	1.09 (0.83–1.44)
WC	Q1 (< 131 g/L)	Q2 (131-136g/L)	Q3 (137-142g/L)	Q4 (≥143 g/L)
	*n* = 499	*n* = 456	*n* = 453	*n* = 483
Incidence, n (%)	238 (47.7)	203 (44.5)	242 (53.4)	246 (50.9)
Model 1	1 (Ref)	0.88 (0.68–1.14)	1.26 (0.98–1.62)	1.14 (0.89–1.46)
Model 2	1 (Ref)	0.90 (0.67–1.17)	1.24 (0.95–1.61)	1.13 (0.87–1.48)
Model 3^‡^	1 (Ref)	0.92 (0.70–1.22)	1.17 (0.88–1.56)	1.02 (0.76–1.37)
BP	Q1 (< 130 g/L)	Q2 (130-136g/L)	Q3 (137-142g/L)	Q4 (≥143 g/L)
	*n* = 509	*n* = 572	*n* = 522	*n* = 512
Incidence, n (%)	216 (42.4)	224 (39.2)	247 (47.3)	224 (43.8)
Model 1	1 (Ref)	0.87 (0.69–1.11)	1.22 (0.95–1.56)	1.06 (0.83–1.35)
Model 2	1 (Ref)	0.81 (0.63–1.05)	1.19 (0.92–1.54)	1.00 (0.77–1.30)
Model 3^§^	1 (Ref)	0.81 (0.61–1.06)	1.20 (0.91–1.59)	0.95 (0.71–1.27)
TGs	Q1 (< 131 g/L)	Q2 (131-137g/L)	Q3 (138-143g/L)	Q4 (≥144 g/L)
	*n* = 835	*n* = 946	*n* = 815	*n* = 847
Incidence, n (%)	142 (17.0)	201 (21.2)	193 (23.7)	205 (24.2)
Model 1	1 (Ref)	**1.32 (1.04–1.67)** ^*^	**1.51 (1.19–1.93)** ^*^	**1.56 (1.23–1.98)** ^*^
Model 2	1 (Ref)	1.23 (0.96–1.58)	**1.49 (1.16–1.91)** ^*^	**1.47 (1.15–1.89)** ^*^
Model 3^¶^	1 (Ref)	1.11 (0.84–1.45)	**1.36 (1.02–1.80)** ^*^	1.05 (0.79–1.41)
HDL-c	Q1 (< 132 g/L)	Q2 (132-138g/L)	Q3 (139-144g/L)	Q4 (≥145 g/L)
	*n* = 566	*n* = 612	*n* = 537	*n* = 556
Incidence, n (%)	114 (20.1)	141 (23)	132 (24.6)	147 (26.4)
Model 1	1 (Ref)	1.19 (0.90–1.57)	1.29 (0.97–1.72)	**1.43 (1.08–1.88)** ^*^
Model 2	1 (Ref)	1.17 (0.88–1.56)	1.27 (0.95–1.70)	**1.46 (1.09–1.93)** ^*^
Model 3^††^	1 (Ref)	1.06 (0.78–1.43)	1.10 (0.80–1.50)	1.14 (0.83–1.57)
FBG	Q1 (< 131 g/L)	Q2 (131-136g/L)	Q3 (137-142g/L)	Q4 (≥143 g/L)
	*n* = 455	*n* = 434	*n* = 449	*n* = 437
Incidence, n (%)	118 (25.9)	106 (24.4)	117 (26.1)	118 (27.0)
Model 1	1 (Ref)	0.92 (0.68–1.25)	1.01 (0.75–1.36)	1.06 (0.79–1.42)
Model 2	1 (Ref)	0.91 (0.66–1.25)	1.06 (0.78–1.44)	1.06 (0.78–1.46)
Model 3^‡‡^	1 (Ref)	0.95 (0.66–1.35)	1.00 (0.69–1.43)	0.94 (0.64–1.38)

Abbreviations: CI, confidential interval; WC, waist circumference; DBP, diastolic blood pressure; SBP, systolic blood pressure; FPG, fasting plasma glucose; HDL-c, high-density lipoprotein cholesterol; TGs, triglycerides; eGFR, estimated glomerular filtration rate; the use of bold emphasis and * are to mark the statistically significant indicators

Model 1 is unadjusted;

Model 2 is adjusted for age, education, smoking and drinking;

Model 3^†^ is adjusted for age, education, smoking, drinking, WC, SBP, DBP, FPG, HDL-c, TGs, uric acid, NAFLD and eGFR

Model 3^‡^ is adjusted for age, education, smoking, drinking, SBP, DBP, FPG, HDL-c, TGs, uric acid, NAFLD and eGFR

Model 3^§^ is adjusted for age, education, smoking, drinking, WC, FPG, HDL-c, TGs, uric acid, NAFLD and eGFR

Model 3^¶^ is adjusted for age, education, smoking, drinking, WC, SBP, DBP, FPG, HDL-c, uric acid, NAFLD and eGFR

Model 3^††^ is adjusted for age, education, smoking, drinking, WC, SBP, DBP, FPG, TGs, uric acid, NAFLD and eGFR

Model 3^‡‡^ is adjusted for age, education, smoking, drinking, WC, SBP, DBP, HDL-c, TGs, uric acid, NAFLD and eGFR

Table 4 shows the risk of developing hyperuricemia according to hemoglobin quartiles. The incidence of hyperuricemia gradually increased with the hemoglobin quartiles in both sexes: 15.6, 18.7, 21.5 and 25.2% in women and 26, 30.4, 33.1 and 33.3% in men in Q1, Q2, Q3, and Q4, respectively. Compared with the first hemoglobin quartile (Q1), the ORs for Q2, Q3, and Q4 were 1.25 (1.00–1.55), 1.49 (1.19–1.86) and 1.83 (1.48–2.26) in women and 1.25 (0.95–1.63), 1.41 (1.08–1.84), and 1.42 (1.08–1.86) in men, respectively. After adjusting for age, education, smoking and alcohol consumption habits, the ORs for Q2, Q3, and Q4 were 1.15 (0.92–1.44), 1.45 (1.15–1.82) and 1.68 (1.35–2.10) in women and 1.24 (0.94–1.64), 1.41 (1.07–1.85) and 1.37 (1.04–1.82) in men, respectively.

**Table 4:** Odds ratio and 95% CI of hyperuricemia at 3-year follow-up according to baseline hemoglobin quartiles

Women	Q1 (< 132 g/L)	Q2 (132-138g/L)	Q3 (139-144g/L)	Q4 (≥145 g/L)
Uric acid	*n* = 1092	*n* = 1226	*n* = 1004	*n* = 1071
Incidence (%)	170 (15.6)	229 (18.7)	216 (21.5)	270 (25.2)
Model 1	1 (Ref)	**1.25 (1.00–1.55)** ^*^	**1.49 (1.19–1.86)** ^*^	**1.83 (1.48–2.26)** ^*^
Model 2	1 (Ref)	1.15 (0.92–1.44)	**1.45 (1.15–1.82)** ^*^	**1.68 (1.35–2.10)** ^*^
Model 3	1 (Ref)	1.00 (0.79–1.27)	1.20 (0.94–1.53)	1.23 (0.97–1.57)
Men	Q1 (< 152 g/L)	Q2 (152-158g/L)	Q3 (159-165g/L)	Q4 (≥166 g/L)
Uric acid	*n* = 535	*n* = 513	n = 522	*n* = 472
Incidence (%)	139 (26.0)	156 (30.4)	173 (33.1)	157 (33.3)
Model 1	1 (Ref)	1.25 (0.95–1.63)	**1.41 (1.08–1.84)** ^*^	**1.42 (1.08–1.86)** ^*^
Model 2	1 (Ref)	1.24 (0.94–1.64)	**1.41 (1.07–1.85)** ^*^	**1.37 (1.04–1.82)** ^*^
Model 3	1 (Ref)	1.19 (0.89–1.58)	1.20 (0 .90–1.60)	1.15 (0.85–1.56)

Abbreviations: CI, confidential interval; the use of bold emphasis and * are to mark the statistically significant indicators

Model 1 is unadjusted;

Model 2 is adjusted for age, education, smoking and drinking;

Model 3 is adjusted for age, education, smoking, drinking, WC, SBP, DBP, FPG, HDL-c, TGs, NAFLD and eGFR

Table 5 presents the risk of new-onset NAFLD according to hemoglobin quartiles. The incidence of NAFLD gradually increased as the hemoglobin quartile increased: 32, 34.6, 38.1 and 39.2% in women and 27.5, 30.7, 34.4 and 36% in men in Q1, Q2, Q3, and Q4, respectively. Compared with the first hemoglobin quartile (Q1), the ORs for Q2, Q3, and Q4 were 1.12 (0.91–1.39), 1.31 (1.06–1.63) and 1.37 (1.10–1.70) in women and 1.17 (0.85–1.60), 1.378 (1.00–1.90, 1.48 (1.07–2.04) in men, respectively. After adjusting for age, education, smoking and alcohol consumption habits, the ORs for Q2, Q3, and Q4 were 1.13 (0.90–1.41), 1.33 (1.06–1.66) and 1.39 (1.10–1.75) in women and 1.15 (0.83–1.60), 1.29 (0.93–1.80) and 1.40 (1.00–1.96) in men, respectively.

**Table 5:** Odds ratio and 95% CI of fatty liver at 3-year follow-up according to baseline hemoglobin quartiles

NAFLD	Q1 (< 131 g/L)	Q2 (131-137g/L)	Q3 (138-143g/L)	Q4 (≥144 g/L)
Women	*n* = 741	*n* = 793	*n* = 711	*n* = 664
Incidence (%)	237 (32.0)	274 (34.6)	271 (38.1)	260 (39.2)
Model 1	1 (Ref)	1.12 (0.91–1.39)	**1.31 (1.05–1.63)** ^*^	**1.37 (1.10–1.70)** ^*^
Model 2	1 (Ref)	1.13 (0.90–1.41)	**1.33 (1.06–1.66)** ^*^	**1.39 (1.10–1.75)** ^*^
Model 3	1 (Ref)	1.19 (0.92–1.54)	1.20 (0.92–1.57)	1.11 (0.84–1.46)
NAFLD	Q1 (< 150 g/L)	Q2 (150-157g/L)	Q3 (158-164g/L)	Q4 (≥165 g/L)
Men	*n* = 356	*n* = 388	*n* = 358	*n* = 331
Incidence (%)	98 (27.5)	119 (30.7)	123 (34.4)	119 (36.0)
Model 1	1 (Ref)	1.17 (0.85–1.60)	**1.38 (1.00–1.90)** ^*^	**1.48 (1.07–2.04)** ^*^
Model 2	1 (Ref)	1.15 (0.83–1.60)	1.29 (0.93–1.80)	**1.40 (1.00–1.96)** ^*^
Model 3	1 (Ref)	0.92 (0.63–1.33)	0.78 (0.53–1.15)	0.90 (0.61–1.34)

Abbreviations: CI, confidential interval; NAFLD, nonalcoholic fatty liver disease; the use of bold emphasis and * are to mark the statistically significant indicators

Model 1 is unadjusted;

Model 2 is adjusted for age, education, smoking and drinking;

Model 3 is adjusted for age, education, smoking, drinking, WC, SBP, DBP, FPG, HDL-c, TGs, uric acid and eGFR

**Fig. 4:**
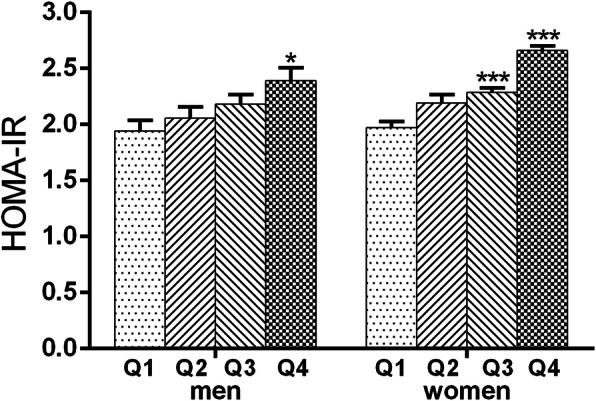
Comparison of HOMA-IR according to different quartiles of hemoglobin concentration Data are presented as mean ± SEM. *p < 0.05, ***p < 0.001 compared to Q1.

In addition, we further analyzed the association of HOMA-IR with different quartiles of hemoglobin. We found that HOMA-IR levels were significantly increased in the highest hemoglobin quartiles (compared to the lowest quartile, *P* < 0.05) in men and in the third and fourth quartiles (compared to the first quartile, *P* < 0.001) in women (Fig. 4).

## Discussion

This prospective follow-up cohort study conducted in a Chinese adult population indicated that a high level of hemoglobin was a potential predictor of MetS incidence in both sexes. To our knowledge, this is the first study to extend these findings to include individual MetS components in a large sample.

The positive association between hemoglobin level and MetS has been reported in cross-sectional studies conducted in various populations, and this association was also detected in other cohort studies. For instance, Laudisio et al. reported that hemoglobin levels were significantly higher in subjects with MetS than in those without MetS [22]. Likewise, Hämäläinen et al. reported that higher hemoglobin levels were related to all components of MetS [6]. Similarly, in our study, we found that the hemoglobin level was markedly higher in participants with MetS or with any of the components of MetS. Moreover, there was a significant association between hemoglobin and cardiovascular risk factors, including BP, WC, TGs, BMI, FPG and serum UA, both in men and women. The data from the present study are consistent with these previously published results [7, 8, 10]. As the number of MetS components increased, the proportion of individuals in the first quartile of hemoglobin decreased, and the proportion of individuals in the fourth quartile increased. This finding further demonstrated an altered hemoglobin level in subjects at high risk of MetS. The mean concentration of hemoglobin was significantly higher in individuals with more MetS components in the study by Ahmadzadeh et al. [8].

One of the most important cohort studies on this topic was conducted by Hashimoto et al. in 2017, which showed that an increased hemoglobin level was associated with the risk of incident MetS in men after an 8-year follow-up period [12]. Another study in the Ethiopian population indicated that this association existed among women but not men [10]. The authors suggested the need to further study this issue. In this study, we found a gradual increase in the incidence of MetS as well as TGs and HDL-c in women and abdominal obesity and HDL-c in men as the levels of hemoglobin increased after adjusting for age, education and lifestyle variables. However, this association did not differ significantly after adjusting for metabolic-related parameters. The findings of a relationship between elevated hemoglobin levels and the risk of developing MetS and its components do not necessarily indicate a direct causal relationship. MetS represents a cluster of simultaneously occurring features; in many cases, the correlations among each of the components are significant. It may be that intervention on one of a pair of components might affect the other. Furthermore, since insulin resistance is the root cause of MetS, each component of MetS exists to some extent with insulin resistance. In this study, hemoglobin was positively associated with insulin resistance, as assessed by HOMA-IR. In addition, differences in ethnicity and race as well as study design differences across study populations may account for the absence of consistency across studies.

Various epidemiological studies have reported the association of hyperuricemia with the components of MetS, pointing out that hyperuricemia could be included in the definition of MetS [23–26]. NAFLD is closely and bidirectionally related to MetS and type 2 diabetes and is currently considered to be the hepatic component of MetS [27, 28]. Our data showed that the risk of developing either hyperuricemia or NAFLD was significantly associated with hemoglobin levels in both women and men. Our data are consistent with emerging literature that demonstrates elevated hemoglobin levels in participants at high risk of NAFLD [29].

Increasing evidence has shown that MetS is associated with an increased risk of CVD and is now considered a secondary target for the treatment of coronary heart disease [2]. Insulin resistance, endothelial dysfunction and inflammation might play crucial roles in the incidence of MetS [30–32]. The possible mechanism linking hemoglobin and MetS might be supported by the pathogenesis described as follows. A high level of hemoglobin can lead to decreased blood flow by increasing blood viscosity and subsequently reduce the delivery of oxygen, glucose and insulin to essential tissues, which might induce insulin resistance [33]. We showed that increased hemoglobin levels are associated with significant insulin resistance, as assessed by HOMA-IR, and there is a strong correlation between hemoglobin and HOMA-IR in both men and women. As increasing quartiles of hemoglobin are associated with more insulin resistance, these data might indicate that the increase in hemoglobin level could be accompanied by an insulin-resistant state.

The alteration of hemoglobin concentration was associated with the pathophysiology of impaired endothelial function [34]. In addition, the hemoglobin concentration was associated with the sCD40L level, which may create a prothrombotic and proinflammatory microenvironment, exacerbating the development of atherosclerosis and MetS [35, 36]. Serum high molecular weight (HMW) adiponectin could improve insulin sensitivity; however, it has been reported that hemoglobin levels are inversely associated with serum HMW adiponectin levels in community-based populations [37]. Furthermore, hemoglobin might upregulate miR-144 expression, leading to an inflammatory response of microglia [38]. Taken together, these findings indicate that the hemoglobin levels are associated with MetS.

The main strength of this study is the relatively large number of subjects stratified by sex both at baseline and at the 3-year follow-up. Lifestyle habits such as smoking and alcohol consumption between participants with and without MetS did not differ significantly. However, the study has several limitations that should be noted. First, the study population was restricted to a middle-aged and elderly population in a rural area in China with a disproportionately high baseline prevalence and incidence of MetS. The specific demographics and data source may have limited the generalizability of the results. It has been demonstrated that iron status is associated with insulin resistance [39]. Unfortunately, we did not measure iron concentrations or serum ferritin, which are both markers of the iron status in an individual. In addition, we did not have complete data on the women’s menopausal status in this study. Additionally, we could not obtain information about the nutritional content of the subjects’ diets or the use of dietary supplements such as antioxidants or iron. Furthermore, the subjects who had chronic diseases such as cancer and malnutrition were not fully excluded, which might have affected various parameters. Finally, the accuracy of the incidence data in the present study might have been notably affected by the relatively brief follow-up period.

As MetS is now recognized as a risk factor for CVDs [40, 41], the association between hemoglobin and MetS might elucidate the high incidence of CVD in patients with MetS. In view of the emerging epidemic status and impact of MetS, the early detection of individuals at high risk for MetS would help prevent associated cardiovascular complications. Furthermore, as hemoglobin concentration is an inexpensive, frequently obtained test in the laboratory, it might potentially be a useful tool in the early identification and evaluation of MetS and CVD prevention and control programs.

## Conclusion

In conclusion, the present study provides additional evidence that hemoglobin plays an important role in predicting new-onset MetS in both women and men. We also demonstrated that hemoglobin is clearly correlated with future risk of low HDL-c, high TGs, hyperuricemia, and NAFLD among women and abdominal obesity, low HDL-c, hyperuricemia, and NAFLD among men. Clarifying the association between hemoglobin level and MetS may provide evidence in support of using readily available, low cost and routinely collected clinical hematological parameters for the early identification of individuals at risk for MetS and CVD. Further large prospective follow-up studies are needed to confirm these findings.

## Data Availability

The data that support the findings of this study are available from REACTION Study Group but restrictions apply to the availability of these data, which were used under license for the current study, and so are not publicly available. Data are however available from the authors upon reasonable request and with permission of REACTION Study Group.
